# Rhinosinusitis derived Staphylococcal enterotoxin B plays a possible role in pathogenesis of food allergy

**DOI:** 10.1186/1471-230X-6-24

**Published:** 2006-08-18

**Authors:** Tao Liu, Bin-Quan Wang, Peng-Yuan Zheng, Shao-Heng He, Ping-Chang Yang

**Affiliations:** 1 Institute of Allergy and Department of Otolaryngology, the First Hospital, Shanxi Medical University, Taiyuan, China; 2 Department of Gastroenterology, the Second Hospital, Zhengzhou University, Zhengzhou, China; 3 Clinical Experimental Center, the First Affiliated Hospital of Nanjing Medical University, Nanjing, Jiangsu, China; 4 Department of Pathology and Molecular Medicine, McMaster University, Hamilton, ON, Canada

## Abstract

**Background:**

Staphylococcal enterotoxin B (SEB) is a potent immunomodulator and implicated with pathogenesis of inflammatory diseases mediated by Th1 or Th2 dominant immune responses. The objective of this study is to determine a possible association between rhinosinusitis derived SEB and pathogenesis of food allergy (FA).

**Methods:**

The study included chronic rhinosinusitis (CRS) patients with FA (N = 46) or without FA (N = 33). Controls included FA patients without CRS (N = 26) and healthy volunteers (N = 25). In CRS patients, we assessed the parameters associated with FA including prick skin test (PST) reactivity to food allergens, serum levels of allergen-specific IgE and cytokines (IL-4, IL-13, IFN-Î^3^), and the number/reactivity of food-allergen specific Th1/Th2 cells in the peripheral blood before and 2 months after sinus surgery. Changes of these parameters were evaluated in comparison with changes in SEB concentration in the sinus lavage and stool samples and also in vitro reactivity to SEB. In CRS patients with FA, we also assessed changes in reactivity to oral challenge of offending food before and after sinus surgery.

**Results:**

Two months following sinus surgery, we observed statistically significant reduction in PST and oral challenge reactivity in CRS patients with FA in parallel to decrease in serum levels of Th2 cytokines (IL-4 and IL-13) and allergen specific IgE. Improvement of reactivity to food allergens was positively associated with decline in SEB concentrations in the sinus lavage and stool samples. In vitro study results also indicated a role of SEB in aggravation of Th2 skewed responses to food allergens. Such changes were not observed in CRS-non FA patients or control FA patients.

**Conclusion:**

The rhinosinusitis derived SEB plays a certain role in the pathogenesis of FA by augmenting and/or maintaining polarized Th2 responses. Removal of SEB-producing pathogens from the rhinosinuses may be beneficial for attenuating the FA symptoms in patients with CRS-FA.

## Background

About 1–2% adults [1] and 4–6% children [2] suffer from IgE mediated food allergy (FA). The clinical symptoms of FA vary from slight abdominal discomfort to life-threatening anaphylactic shock. Avoidance of offending food is the only effective therapeutic measure currently. FA are prevalent irrespective with sex, ethnicity, age, and social/economic class and imposes a heavy burden on health care expenditure and global economy [3].

Although etiology of FA is still not well understood, significant progress has been made for the recent few years with regard to molecular mechanisms of FA [4]. IgE mediated food allergy is believed to be mediated by type 2 T helper (Th2) cell responses to food allergens. Th2 cytokines, such as IL-4 and IL-13, promote food allergen-specific IgE production. IgE-dependent mast cell activation is regarded as the basis of clinical symptoms of food allergy [5]. However, the initial steps of antigen specific IgE over production in the intestine remain to be elucidated. Specific T cell clones to specific antigens are generated during sensitization. It is unclear what factors dictate naïve T helper cells to differentiate to antigen specific Th2 cells; how antigen specific immune memory cells live for a long time and keep bioactivity in the body. It has been proposed that multiple factors are involved in the development and severity of food allergy including genetic, environmental and immunological factors [6], but the contributions of these factors to FA pathogenesis remain poorly understood.

Staphylococcus aureus-derived enterotoxin B (SEB) is one of the superantigens that possess potent stimulatory activity on T lymphocytes [7,8]. Antigen-independent, polyclonal T cell activation by SEB can worsen inflammatory condition in both Th1 mediated and Th2 mediated diseases including chronic rhinosinusitis. Thus controlling staphylococcal colonization or infection may have therapeutic implications in these conditions. It is reported that prophylactic antibiosis for *S. aureus *revealed protective effects in atopic dermatitis. However, a role of superantigens in FA is not well understood [9-11].

In clinical practice, we have noticed that some patients with both chronic rhinosinusitis (CRS) and FA demonstrate significantly amelioration of FA after treatment of CRS that can not be explained with the primary treatment. Our previous studies in rodents revealed that concurrent oral administration of SEB and food antigens (by gavage feeding) induces IgE-mediated reactivity to food antigens in intestinal mucosa [12]. We hypothesized that CRS-derived microbial products (such as SEB) in nasal discharges could be swallowed down the gastrointestinal tract to disturb the immune homeostasis in the local tissue. If our hypothesis is correct, attenuation of FA symptoms is likely to be observed in CRS-FA patients following sinus surgery secondary to reduction of SEB swallowed to the GI tract. We thus examined changes in food allergen reactivity to PST/oral challenge along with other parameters of FA (serum cytokine levels, serum allergen specific IgE, and allergen specific Th2 cells) following sinus surgery in CRS-FA patients in comparison with SEB concentration in the sinus and stool and SEB reactivity.

## Methods

### Subjects

This prospective study was carried out at the Institute of Allergy and Department of Otolaryngology, Shanxi Medical University. Patients with both CRS and FA were selected to this study (These patients had had CRS first, then FA). The study was approved by the medical ethics committee at Shanxi Medical University. Informed written consent was obtained from each patient. The demographic data of the subjects included in this study were presented in Table 1.

### Diagnosis of food allergy

#### Case history

The case history of food allergy was provided by the patients. Symptoms after specific foods ingestion included a tingling sensation in the mouth, swelling of the tongue and the throat, difficulty breathing, hives, vomiting, abdominal cramps, diarrhea, drop in blood pressure, loss of consciousness, etc. Symptoms typically appear within minutes to two hours after the ingestion of the food to which he or she is allergic.

#### Skin prick test

One drop of extract of each fresh food (purchased from Beijing Xiehe Hospital, Beijing, China) was applied to the patients' forearm. The skin covered by the drop of food antigen was pricked with 1 mm single-peak lancets. Positive control was performed using 10 mg/ml histamine dihydrochloride; the negative control using saline solution. The results were read after 15 min. The outline of each weal was documented using a piece of transparent paper to copy the wheal [13]. The skin wheal areas were determined with scanned digital files using the software ImageJ. Skin prick tests were regarded positive when the area was larger than 7 mm^2^. Subjects with a weal area below 3 mm^2 ^in positive controls were excluded from this study.

#### Oral challenge with food antigens

Oral challenges were performed in all the subjects in this study using one of the food allergens that the study subject revealed positive prick skin test reactivity (usually using the one had the largest wheal area). We used 10 common food allergens in the skin prick test to screen the offending allergens in these patients including cow milk, hen eggs, wheat, soy, shrimp, peanut, carrot, pork, beef, and fish although some patients might also sensitize to other food antigens. Patients being treated with an antihistamine or corticosteroids were advised to stop taking it for 72 h before provocation. The oral challenge was performed in double blind manner. The antigen extracts or placebos were contained in coded capsules. The patients took 3 capsules of antigen or placebo; they were asked to take another 3 capsules (placebo or antigens) one week later. The study subjects were closely monitored for 4 hours following oral challenge in the facility where treatment of anaphylaxis was readily available. The healthy controls and CRS patients were also oral challenged with a mixture of the 10 antigens. The clinical symptoms were scored as 0 to 5. Score 5 indicated severe; score 0 indicated no symptom.

#### Serum levels of cytokine and allergen-specific IgE

Blood samples were collected from each patient before and two month after the treatment of CRS, and also from healthy controls and CRS alone patients with two months apart from each time. The sera were separated immediately after withdrawn and stored at -70°C until use. The levels of total IgE, antigen specific IgE, IL-4, IL-13 and IFN-γ in the sera were measured with ELISA. The reagent kits of IgE were purchased from Pharmacia (Beijing, China.) The reagent kits of cytokines were purchased from Genetimes Tech Inc. (Shanghai, China). The procedures were following the manufacturer's instructions. Negative controls included omitting the primary antibodies and using isotype IgG instead of the primary antibodies for each parameter.

### Diagnosis and treatment of CRS

We diagnosed CRS as inflammation of the sinus mucosa with a persistent mucoid or mucopurulent nasal discharge for longer than 3 months that resisted antimicrobial therapy and antral irrigation. Diagnosis was made on the basis of clinical history, rhinoscopic findings, and computed tomographic scan of rhinosinuses. CRS was confirmed by computed tomographic examination that showed diffuse mucosal thickening in the ethmoid or/and maxillary sinuses bilaterally with scores higher than 12 by the Lund-Mackay staging system [14]. The procedures of FESS (functional endoscopic sinus surgery) followed the previous reports [15]. The surgical side of sinus was irrigated daily post-FESS until the irrigated fluids became clear. After discharge, the patients were required to visit the hospital to conduct a post-operation examination of nasal cavity and sinuses on a monthly basis for the first year. Any unusual feelings in the operated sinus area were recorded. Physicians would do necessary treatment for relapse of inflammation in the sinuses during the entire follow-up period.

The CRS in the patients selected in this study had been treated with many means and got poor results. They thus pleaded to FESS following physician's advice. All the CRS patients (including CRS-FA and CRS alone) underwent FESS in their maxillary sinusitis. Among the CRS-FA patients, 8 (17.4%) patients also underwent bilateral ethmoidectomy. 9 patients also underwent unilateral ethmoidectory (19.6%, right or left side). 6 (13%) patients also underwent septum orthomorphia. 18 (39.1%) patients had nasal polyps that were removed during FESS. 3 (6.5%) patients underwent partial middle turbinectomy. 6 (13%) patients underwent inferior turbinectomy. During the 2 month observation period, all the patients underwent regular follow-up visit (The total follow-up period lasted for one year after FESS). 2 months after FESS, all the patients reported significant improvement in their clinical symptoms of CRS. Clinical symptom scores were recorded before and 2 months after FESS that were defined from 0 to 4 (0 for no symptoms; 4 for severe). Most patients had moderate to severe CRS clinical symptoms before FESS; 40 out of 46 (87%, scored 0 and 1) patients showed significant attenuation in their clinical symptom of sinusitis.

### Measurement of SEB in sinus (or nasal) wash fluids (SWF) and stools

Every patient with CRS underwent maxillary sinus puncturing and irrigating with saline. The SWF was collected prior to other procedures. Five ml saline was injected into the sinus cavity and recollected and stored at -70°C for further analyses. For the ethic reason, nasal lavage fluids (5 ml saline) instead of sinus irrigation were collected from the healthy controls and FA controls. None of the subjects had acute upper respiratory acute infections within the past month. Stool samples were also collected. 100 mg stool samples were dissolved in 0.5 ml PBS, mixed well, stayed at 4°C for 20 min, centrifuged for 10 min at 17,000 × g at 4°C. The supernatants were collected and subjected to the evaluation of SEB, SEA and TSST1 (toxic shock syndrome toxin 1). Contents of SEB, SEA and TSST1 in SWF were evaluated with ELISA following reagent manufacturer's instructions.

### Identification of S. aureus in sinuses

Surgically removed sinus tissues were obtained from each patient (a nasal lateral wall swab was obtained from FA alone patients and healthy controls instead) and were sent to the Microbiological Laboratory within 30 minutes. 100 mg mucosa was homogenized in 0.1 ml saline. The same volume of the homogenates from each patient was cultured aerobically on blood agar plates. Colonies of different morphology growing on the agar were separately enumerated, subcultured on blood agar, Gram stained, and tested for catalase production. Isolates with a typical Gram-stained appearance and a positive catalase test were tested for coagulase production, and positive isolates were regarded as *S. aureus*. The template DNA was extracted from the positive cultured colony of each subject with the procedures reported by Riffon [16]. PCR was performed with the samples from each subject. The primers of SEB were referred to the previous report [17]: 5'-ttgcatatccgcgtcaaata-3' and 5'-cttcatgttctttcgcatcg-3'. Amplification was performed on a Perkin-Elmer (Norwalk, Conn.) thermocycler in 25-μl reaction mixtures. The program consisted of an initial denaturation step at 94°C for 2 min, followed by 22 cycles of 1 s at 94°C, 2 s at 58°C, and 10 s at 72°C, with a final extension step at 72°C for 5 min. Amplification products were separated on a 1% agarose gel and stained with ethidium bromide before being analyzed on a UV bench by using GelDoc2000 (Bio-Rad). The PCR products were routinely sequenced to confirm amplification of the targeted sequence.

### Antigen specific CD4 T cell proliferation assay

Heparinized blood samples (40 ml) were obtained from each subject before the sinus surgery. PBMCs were separated by gradient density centrifugation. 5 × 10^5^/ml PBMCs were cultured in vitro in the presence or absence of a food allergen to which the study subject revealed the most significant prick skin test reactivity (a mixture of the 10 antigens was used in the culture of PBMCs from the controls) at a dose of 10 μg/ml for 96 h. A portion of the cells were cultured with SEB (20 μg/ml) in addition to the specific antigens. The supernatants were collected at the end of the culture. The cytokines of Th1 (IFN-γ) and Th2 (IL-4) in the supernatants were determined with ELISA as described above. Another batch of cells were cultured in the presence of brefeldin A (10 μg/ml, Sigma) in addition to the culture conditions above. These cells were fixed with 1% paraformaldehyde for 1 h at room temperature, stained with FITC labeled anti-IL-4 antibody (for Th2 cells) and PE labeled anti-IFN-γ antibody (for Th1) for 30 min on ice. The positively stained cells were counted with flow cytometry.

Another batch of PBMCs from patients with CRS-FA were cultured in the presence of the specific antigens and SEB (20 μg/ml) or in the presence of specific antigen (or SEB) alone. The supernatants were sampled on day 0 and every 3 days. IL-4 levels in the supernatants were measured with ELISA as described above.

### Statistics

Outcome variables were summarized descriptively using means and standard deviations for continuous variables and counts and proportions for categorical variables. A paired T-test for continuous variables and McNemar's Test for binary variables was used to evaluate the within group differences (between the first and the second measurements or between the measurements taken before and after the sinus surgery). Between-group differences in the clinical and laboratory characteristics in patients with CRS-FA and healthy control or patients with FA or with CRS were analyzed using Kruskal-Wallis test or ANOVA as appropriate. Post-hoc comparisons were performed using Mann-Whitney U test or Student t test to look for significant differences between pairs of subject groups. The correlations between the reduction of individual antigen induced clinical symptom score after FESS and other parameters were determined by Spearman rank correlation coefficient respectively. All comparisons were made two-sided, and p < 0.05 was considered significant.

## Results

### FESS attenuated reactivity to oral food allergen challenge in CRS-FA patients

Each subject underwent two times of skin prick test with the 10 designated antigens before and two months after the sinus surgery. The healthy control group did not show any positive reactions to the 10 antigens (the wheal area was less than 7 mm^2^), nor in the patients with CRS alone. The wheal area larger than 7 mm^2 ^was observed in the CRS-FA patients in 2 to 8 antigens that was markedly decreased in two months after the FESS (Table 2). Two months following FESS, CRS-FA patients revealed significant reduction of FA symptoms provoked by oral challenge of offending food allergens. There was no change in reactivity in FA patients in the repeated oral challenge test. FESS reduced serum levels of Th2 cytokines and allergen specific IgE in CRS-FA patients (Table 3).

### Treatment of CRS attenuated clinical symptoms of FA

As shown in Table 4, both CRS-FA patients and FA patients showed gastrointestinal symptoms in response to the food antigen challenge. A mixture of the 10 food antigens using in this study was taken in by the healthy controls and the CRS controls; none of them reported any gastrointestinal symptoms. The placebo did not evoke any symptoms in all the subjects. Two months after the FESS, the oral antigen challenge was repeated. The clinical symptoms induced by the first oral antigen challenge were significantly attenuated in patients of CRS-FA group but not in FA group (Table 4).

### Levels of serum Th2 cytokines and specific IgE decreased after CRS treatment

**Figure 1:**
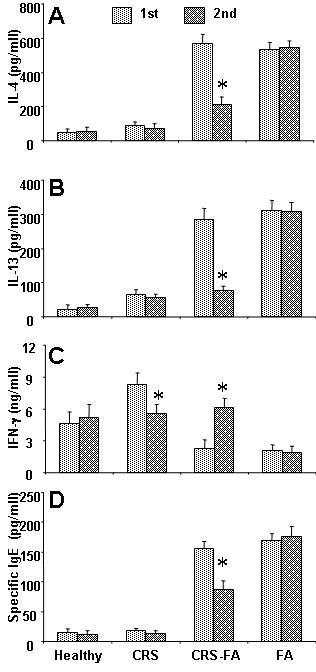
Th2 cytokines and IgE levels were decreased in the sera of patients with CRS-FA after the sinus surgery. Th1/Th2 cytokines and specific IgE were measured with ELISA as detailed in the text. Bars represent levels of cytokines or IgE in the sera. Data are expressed as mean ± SD. *, p < 0.05, compared with values of the 1^st ^tests (before surgery).

Significant reduction of serum levels of Th2 cytokines (Fig 1A, B) and antigen specific IgE (Fig 1D) were observed in patients with CRS-FA 2 month after the FESS, but no changes in the serum levels of IFN-γ were observed (Fig 1C). In patients with CRS alone, the Th1 pattern cytokine levels were attenuated after the FESS (Fig 1C), but their serum Th2 cytokines did not change significantly (Fig 1A, B), nor did the specific IgE levels (Fig 1D). We did not notice much change in the serum cytokine and specific IgE levels in healthy controls between the two tests (Fig 1A–D).

### SEB was identified in the sinus lavage and stool samples in CRS-FA patients at high concentration

**Figure 2:**
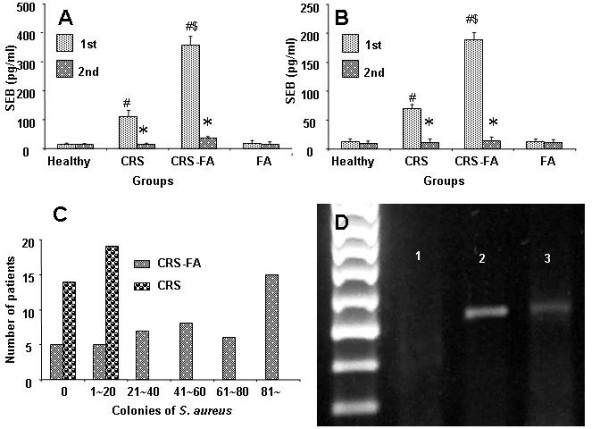
SEB was detected in the sinuses and in the stools of CRS-FA patients. Bars represent SEB levels in the sinus (or nasal) wash fluids (A) or in the stools (B) that were detected with ELISA. Data are expressed as mean ± SD. *, p < 0.05, compared with values of the 1^st ^tests (before the sinus surgery; 2^nd^: 2 months after the sinus surgery). #, p < 0.05, compared with healthy controls. $, p < 0.05, compared with CRS patients. C, *S. aureus *was identified with the surgical sinus samples after bacterial culture. D, *S. aureus *DNA was identified with PCR. Band 1 is the negative control; bands 2 and 3 are the representative bands of samples from patients with CRS-FA.

The levels of SEB, but not SEA and TSST-1 were detected in sinus wash fluids and in the stools that were significantly higher in patients with CRS-FA as compared to patients with CRS alone or to healthy controls (Fig 2A and 2B). Bacterial culture resulted in the *S. aureus *growth in surgically removed sinus tissue in 41 out of the 46 patients (89.1%) with CRS-FA. The numbers of *S. aureus *colonies in the culture ranged from 0 to 96 with an average of 27.8/patient. Much less number of *S. aureus *was detected in surgical removed sinus tissues of patients with CRS only (63.63%) ranged from 0 to 20 colonies per patient (Fig 2C). PCR results showed the *S. aureus *DNA amplified products from surgical removed tissue of 46 (100%) patients that further confirmed the existence of *S. aureus *infection in the sinuses of patients with CRS-FA (Fig 2D). PCR amplified product sequence analysis confirmed that the amplified bands were in consistent with the target DNA sequences.

### Food allergen-specific Th2 responses are augmented by SEB in vitro in CRS-FA patients but not in FA patients

**Figure 3:**
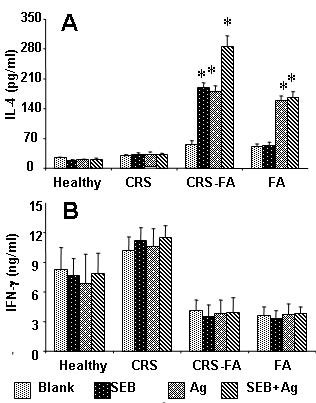
Peripheral blood mononuclear cells (PBMCs) were got from all subjects and cultured in the presence of specific antigens. Supernatants were collected on day 4 and subjected to the assessment of IL-4 (A) and IFN-γ (B) with ELISA. Blank: no special treatment. Ag: treated with specific antigens. SEB: treated with SEB; SEB+Ag: treated with the combination of SEB and Ag. Data are presented as mean ± SD. *, p < 0.05, compared with blank group; #, p < 0.05, compared with the groups treated with Ag alone or with SEB alone.

When cultured with food allergens or SEB, PBMCs from CRS-FA patients produced higher levels of IL-4 (Fig. 3-A, Table 5) than cells cultured without stimuli. PBMCs stimulated with food allergen plus SEB revealed the highest IL-4 production. PBMCs from FA patients also increased the IL-4 production in response to stimulation of food allergens. Food allergen-specific Th2 responses are augmented by SEB in vitro in CRS-FA patients but not in FA patients.

**Figure 4:**
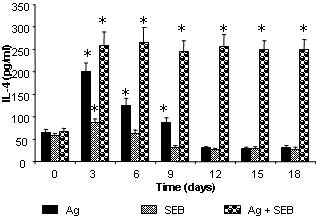
Separated PBMCs from patients with CRS-FA before FESS were cultured in the presence of specific antigens and SEB or antigen (or SEB) alone. Supernatants were sampled every three days and subjected to IL-4 analysis. Bars represent IL-4 levels in the supernatants (mean ± SD). *, p < 0.05, compared with IL-4 levels at day 0 within the same group.

In order to determine if SEB has any effect in maintaining the immune activity of antigen specific Th2 cells in CRS-FA patients, separated PBMCs were cultured with specific antigens in the presence or absence of SEB or SEB alone. In consistent with previous reports [18,21], we observed the rapid increases in IL-4 production in samples from patients with CRS-FA. However, the IL-4 production declined rapidly afterwards in PBMCs exposed to antigen alone or SEB alone. The IL-4 production kept at high levels through the observed period in the PBMCs exposed to both specific antigens and SEB concurrently (Fig 4).

The correlations between reductions of individual antigen induced clinical symptom score after FESS and other 12 parameters collected before FESS were determined by Spearman rank correlation coefficient respectively. As shown in Table 6, FESS, SEB and *S. aureus *were significantly correlated with the reduction of antigen induced clinical symptom scores.

## Discussion

Rhinosinusitis is one of the most common health care complaints in both industrial [19] [48] and developing countries [20] [49]. *S. aureus *is a common pathogen that contributes to both acute and chronic rhinosinusitis [21] [50]. The cumulative evidence indicates that SEB is involved in the pathogenesis of allergic diseases [22] [51]. The coincidence of CRS and food allergy has been noticed by us from late of 1980s as well as by others [23,24] [52, 53]. The results of our study revealed evidence of a role of SEB produced as a result of CRS for development and/or aggravation of Th2 responses to food allergens in the gut mucosa.

The sinus surgery has resulted in an attenuation of Th2 skewed responses to food allergens in CRS-FA patients, most likely secondary to removal the source of SEB. Because of opening to natural environment, the sinuses are easily to become contaminated and colonization by *S. aureus*. Clinical evidence has indicated that *S. aureus *is a common pathogen in acute and chronic rhinosinusitis [25,22]. The rhinosinusitis secretions (some contain SEB) are naturally discharged to the nasal cavity and compelled backward by the ciliated nasal mucosa, sometimes to be swallowed down the gastrointestinal tract. This postulation is supported by the present results. The results showed that *S. aureus *colonization in the sinus mucosa was more than 90% patients with CRS-FA; high levels of SEB in the sinus wash fluids and in the stools were detected. Others also have identified *S. aureus *in human intestine [26-28,23-25]. SEB is an immune active molecule that can affect the intestinal immune homeostasis. Previous studies indicate that SEB can bind, without processing by antigen processing cells, to an outside domain of major histocompatibility class II molecules and the variable portion of the β-chain of the T-cell receptor on specific subsets of T cells, and so can activate up to 25% of all T cells [29,26]. In another word, SEB can induce polyclonal T cell activation, leading to aberrant immune responses. Our recent study shows that SEB compromises intestinal epithelial barrier function and increases intact protein antigen absorption [30,20]. These intact antigens thus have the opportunity to contact the immune cells in the intestinal mucosa and further compromise the intestinal immune homeostasis. The present data are in consistent with our previous studies which demonstrate that SEB acts as an adjuvant in inducing intestinal sensitization in a murine model [30,20] and is associated with ulcerative colitis [17].

Antigen skin test is an initial approach in diagnosing FA. Previous reports have mentioned a positive correlation between IL-4 positive mast cells and the wheal size [31,27]; antigen specific IgE levels and cutaneous allergic reactions [32,28]. The attenuation of wheal size of skin prick tests implicates that less number of IgE molecules has been produced and bound to mast cell surface. FESS appeared to have significantly attenuated prick skin test reactivity to food allergens.

Inhibition of FA clinical symptoms is the summit aim of FA research and clinical practice. The present study documented a significant amelioration of FA clinical symptoms from specific antigen challenge in CRS-FA patients after the sinus surgery. In line with previous reports [33,34,30,31], the results also showed the high levels of serum Th2 cytokines and antigen specific IgE in the FA patients in this study. The improvement occurred in the CRS-FA patients after the sinus surgery, but not in the FA alone patients, implicates that the sinus surgery has resulted in immune regulatory effect, possibly attributing to the removal of pathogen from the sinuses.

As reported before, we also examined *S. aureus *infection in the sinuses of these CRS-FA patients. As expected, more than 90% patients had *S. aureus *colonization in their sinuses. The removal of *S. aureus *infection from the sinuses positively correlates with the attenuation of FA clinical symptom. This event revealed a possible association between the *S. aureus *infection in the sinuses and the pathogenesis of FA. SEB has high immune activity in activating T cells and induces aberrant immune reactions. It is true that most reports on the topic of SEB discuss its effect on Th1 pattern reaction [35,36,32,33]. Yet the cumulative evidence demonstrates that SEB is also involved in the disorders of Th2 reactions such as allergic dermatitis [37,34], allergic rhinitis, asthma [38,35], etc as observed clinically. Many investigators observe the effect of SEB alone on immune function. In reality, in most situations, molecules act with one another concurrently or synergistically in the body. When animals were exposed to SEB and food antigen ovalbumin concurrently as we reported previously [17], intestinal sensitization was induced whereas those animals exposed to SEB or ovalbumin alone did not show any signs of intestinal sensitization, implicating that SEB may act as an adjuvant in the induction of intestinal sensitization with food antigens. SEB can cause polyclonal T cell activation and depending on the pre-existing inflammatory condition, either Th1- or Th2- mediated inflammatory condition can be aggravated.

In general, the re-expose of atopic patients to specific allergens provokes a Th-2 response, with increases in the production of IL-4 and IL-13. These cytokines facilitate IgE production. The CRS-FA patients in this study showed an aberrant Th2 pattern immune reaction as high levels of serum antigen specific IgE, IL-4, IL-13 and low levels of IFN-γ were recorded before the treatment of their sinus disorders (Fig 3). This is in consistent with the concept that food allergy belongs to Th2 immune disorder [30,33]. The attenuation of Th2 cytokines and IgE antibody after the sinus surgery in these patients would suggest an improvement of the aberrant Th2 polarization; although the precise mechanism needs to be further elucidated, the results demonstrate a possibility that this improvement can be associated with reduction of *S. aurus *colonization after FESS.

One of the approaches we took in this study is the exposure of separated PBMCs to specific antigens and SEB. A rapid increase in the IL-4 production as well as the antigen specific Th2 cell proliferation was observed *in vitro*. The phenomenon is well documented in the skewed Th2 polarization disorders such as FA [39,38] and asthma [40,39]. Our results support the notion that the antigen specific Th2 cells exist in the peripheral blood stream of the patients with FA. These cells are capable of rapid proliferating to effector cells in response to stimulation of specific antigens. The addition of SEB to the culture further increased Th2 responses in the PBMCs from patients with CRS-FA, but not from those with either CRS or FA alone. The presented data indicate that SEB may further augment responses of food allergen specific Th2 cells directly or by bystander effect. This point might be the basis that SEB and specific antigens can act synergistically on the immune memory T cells to augment their functions. Similar results have been reported in patients with allergic rhinitis and nasal carriage of *S. aureus *[41,40]. SEB has the property to modulate the T cell immunity via inducing polyclonal T cell expansion, or deletion or anergy depending on certain environment [42,41]. The CRS-FA patients might have genetic predisposition to respond rigorously to SEB and by bystander effect, their Th2 responses to food allergens may be further augmented as one possible explanation. There may be other possibilities as well. After the removal of SEB source, the pathogen from the sinuses, the FA signs, including the wheal area in skin prick tests and the clinical symptoms evoked by exposure to specific food antigens are significantly attenuated. The data from cell culture show that SEB is required in maintaining the immune activity of antigen-specific Th2 cells from CRS-FA patients further supporting the clinical findings. It is likely the FA status in these patients relies on the existence of SEB, at least partially.

## Conclusion

In summary our study revealed significant improvement of reactivity to food allergens by PST and oral challenge in CRS-FA patients after FESS. Improved FA symptoms are paralleled with evidence of attenuated Th2 responses to food allergens and decrease of SEB concentrations in the sinus and the gut. Along with in vitro study results indicating augmenting action of SEB on food allergen specific Th2 responses in CRS-FA patients, the presented data support a role of SEB in development/aggravation of FA in some CRS patients.

## Abbreviations

CRS, chronic rhinosinusitis; FA, food allergy; SEB, Staphylococcal enterotoxin B; FESS, functional endoscopic sinus surgery; PBMC, peripheral blood mononuclear cell; IL, interleukin.

## Competing interests

The author(s) declare that they have no competing interests.

## Authors' contributions

PCY was involved in study design, a portion of FESS, histology, data analysis, some experiments and manuscript preparation; TL and BQW were involved CRS treatment, FESS and experiments; PYZ and SHH were involved as consultants in FA management.

## Pre-publication history

The pre-publication history for this paper can be accessed here:



## Figures and Tables

**Table 1:** Demographics of the study subjects

Groups		CRS	FA	CRS-FA	Healthy
Sex	Female	17	13	26	12
	male	16	13	20	13
Age	Range	22~43	17~46	16~55	20~22
	Average	24.6 ± 5.2	28.6 ± 3.9	25.8 ± 4.8	20.3 ± 0.3
FA history (years)	Range	No	0.8~11	0.5~11	No
	Average	No	3.1 ± 2.6	3.3 ± 2.1	No
CRS history	Range	2.5~11	No	2.2~18	No
	Average	6.3 ± 2.4	No	5.4 ± 1.7	No
Other allergy		No	No	8 with AD	No
Previous FESS		No	No	No	No
Polyposis		8	0	18	0
hyperplastic sinusitis		6	0	12	0
Serum total IgE (pg/ml)		76.9 ± 21.8	699.3 ± 85.2*	765.2 ± 94.3*	55.6 ± 15.4

*, p < 0.05, compared with healthy group. FA: food allergy. CRS: chronic rhinosinusitis. AD: atopic dermatitis. FESS: functional endoscopic sinus surgery.

**Table 2:** Wheal area (mean ± SD, mm^2^) of skin prick test

	Healthy	CRS	CRS-FA	FA
1st	2.1 ± 1.2	5.5 ± 1.8	89.8 ± 14.5	75.3 ± 8.9
2nd	2.2 ± 1.2	4.2 ± 1.2	23.6 ± 5.6*	81.2 ± 12.2

1^st^: the first skin prick test; 2^nd^: the second skin prick test.

*, p < 0.05, compared with the results of the first skin prick test within the same group.

**Table 3:** Positive antigen number (mean ± SD) of skin prick test

Skin Test	CRS-FA	FA
1^st^	7.3 ± 1.1	6.8 ± 0.6
2^nd^	3.2 ± 0.8*	6.7 ± 0.5

1^st^: the first skin prick test; 2^nd^: the second skin prick test.

*, p < 0.05, compared with the results of the first skin prick test within the same group.

**Table 4:** Clinical symptom scores (mean ± SD) evoked by oral antigen challenge

Patients	Challenge	Nausea	Vomiting	Diarrhea	Gut cramp
CRS-FA	1^st^	4.1 ± 0.6	2.3 ± 0.3	1.9 ± 0.2	2.9 ± 0.4
	2^nd^	1.1 ± 0.3*	1.1 ± 0.3*	0.6 ± 0.2*	0.8 ± 0.3*
FA	1^st^	4.2 ± 0.7	2.3 ± 0.5	1.6 ± 0.4	2.8 ± 0.4
	2^nd^	4.3 ± 0.8	2.4 ± 0.8	1.5 ± 0.5	2.5 ± 0.5

1^st^: the first oral antigen challenge; 2^nd^: the second oral antigen challenge.

*, p < 0.05, compared with the results of the first skin prick test within the same group.

**Table 5:** Frequency of IL-4+ or IFN-γ+ T cells following stimulation with SBE, food allergen, or both (%, assessed by flow cytometry)

Treatment	Th1 or Th2	Healthy	CRS	CRS-FA	FA
Blank	Th1	23.3 ± 5.4	34.3 ± 4.2	11.4 ± 4.4	13.4 ± 2.5
	Th2	25.3 ± 3.4	18.6 ± 2.5	35.3 ± 3.3	35.6 ± 2.6
SEB	Th1	20.8 ± 3.6	30.8 ± 4.3	10.3 ± 2.1	10.7 ± 0.6
	Th2	22.3 ± 4.1	15.3 ± 2.2	45.8 ± 5.2*	12.5 ± 1.3*
Ag	Th1	20.2 ± 4.2	35.2 ± 3.2	10.2 ± 1.6	10.2 ± 1.1
	Th2	26.3 ± 6.4	16.6 ± 3.3	46.9 ± 2.8*	36.7 ± 3.3
SEB + Ag	Th1	20.7 ± 3.6	33.8 ± 2.5	5.8 ± 0.3*	10.6 ± 0.5
	Th2	21.9 ± 5.1	15.6 ± 4.5	81.9 ± 6.7*#	31.2 ± 2.9

Data are presented as mean ± SD. *, p < 0.05, compared with the blank cells. #, p < 0.05, compared with the cells treated with SEB or Ag alone. Comparison was done between treatments within the same group. IL-4 was detected to present Th2 profile and IFN-γ was detected to present Th1 profile.

**Table 6:** Univariate analysis on reduced antigen induced clinical symptom score as a dependent variable

Independent variable	r	p
Ag	-0.061	NS
Sex	0.052	NS
Case history (CRS)	0.101	NS
Case history (FA)	0.118	NS
S. aureus colonies	0.696	<0.01
SEB (sinus)	0.659	<0.01
SEB (stool)	0.797	<0.01
FESS	0.889	<0.01
Wheal area of skin prick test	0.029	NS
Serum specific IgE	0.036	NS
Serum IL-4	0.057	NS
Serum IL-13	0.048	NS
Serum IFN-gamma	-0.059	NS
